# Irisin Concentrations in Children and Adolescent Cancer Survivors and Their Relation to Metabolic, Bone, and Reproductive Profile: A Pilot Case–Control Study

**DOI:** 10.3390/jcm14145098

**Published:** 2025-07-17

**Authors:** Despoina Apostolaki, Katerina Katsibardi, Vasiliki Efthymiou, Charikleia Stefanaki, Aimilia Mantzou, Stavroula Papadodima, George P. Chrousos, Antonis Kattamis, Flora Bacopoulou

**Affiliations:** 1 Center for Adolescent Medicine and UNESCO Chair in Adolescent Health Care, First Department of Pediatrics, Medical School, National and Kapodistrian University of Athens, Aghia Sophia Children’s Hospital, 11527 Athens, Greece; ap_desp@yahoo.gr (D.A.); fbacopoulou@med.uoa.gr (F.B.); 2 Division of Pediatric Hematology-Oncology, First Department of Pediatrics, Medical School, National and Kapodistrian University of Athens, Aghia Sophia Children’s Hospital, 11527 Athens, Greece; katharinakats@hotmail.com (K.K.); ankatt@med.uoa.gr (A.K.); 3 University Research Institute for the Study of Genetic and Malignant Disorders in Childhood, Medical School, National and Kapodistrian University of Athens, 11527 Athens, Greece; chrousos@gmail.com; 4 Division of Endocrinology, Metabolism, and Diabetes, First Department of Pediatrics, Medical School, National and Kapodistrian University of Athens, Aghia Sophia Children’s Hospital, 11527 Athens, Greece; amantzou@med.uoa.gr; 5 Department of Forensic Medicine and Toxicology, Medical School, National and Kapodistrian University of Athens, 11527 Athens, Greece; stpapd@gmail.com

**Keywords:** irisin, cancer survivors, childhood cancer survivors, children, adolescents, parathyroid hormone, PTH

## Abstract

**Background/Objectives**: Childhood cancer survivors (CCS) experience chronic health problems and significant metabolic burden. Timely identification of CCS at higher metabolic risk requires novel biomarkers. Irisin, a novel myokine/adipokine has been associated with metabolic, bone and reproductive diseases, but its role in the health of CCS is unknown. The aim of this study was to examine irisin concentrations in children and adolescent CCS (vs. controls) and their association with metabolic, bone and hormonal parameters. **Methods**: Children and adolescent CCS, aged 8–18 years, as well as healthy controls, underwent a detailed physical, body composition, biochemical, hormonal and serum irisin assessment at least 6 months post-treatment. **Results**: A total of 59 children and adolescents (36 CCS, 23 controls; mean age ± SD 12.8 ± 2.9 years; 10 prepubertal, 49 pubertal) participated in the study. Serum irisin concentrations (ng/mL) were significantly lower in CCS than controls [median (IQR) 6.54 (4.12) vs. 11.70 (8.75) ng/mL, respectively, *p* < 0.001]. In the total study sample, serum irisin was correlated negatively with LH (r_s_ = −0.314, *p* < 0.05), CRP (r_s_ = −0.366, *p* < 0.005), age (r_s_ = −0.323, *p* < 0.05) and positively with ALP (r_s_ = 0.328, *p* < 0.05). Serum irisin was also positively correlated with ApoB and Lpa (r_s_ = 0.410 and 0.421, respectively, *p* < 0.05) in CCS, and with PTH (r = 0.542, *p* < 0.005) in controls. Multivariate linear regression analysis indicated parathyroid hormone (PTH) as the only independent variable affecting irisin concentrations. **Conclusions**: Study results reinforce the irisin–PTH interplay hypothesis. Future studies are needed to clarify the potential role of irisin as a bone biomarker of CCS in childhood and adolescence.

## 1. Introduction

The American Cancer Society uses the term cancer survivor to refer to anyone who has ever been diagnosed with cancer no matter where they are in the course of their disease [1]. An estimated number of 15,000 children and adolescents aged 0 to 19 years are diagnosed with cancer each year in the USA, and more than 85% survive for at least 5 years [2,3].

Up to the age of 14 years the most common types of cancer are central nervous system (CNS) tumors (26%) followed by acute lymphoblastic leukemia (21%). In adolescents aged 15 to 19 years, the most common types of cancer include CNS tumors (21%), thyroid cancer (12%), Hodgkin lymphoma (11%), and germ cell and gonadal tumors (10%) [3]. Other types of childhood cancer include non-Hodgkin lymphoma, acute myeloid leukemia, sarcomas, neuroblastoma, Wilms tumor, retinoblastoma, and hepatoblastoma. By the age of 45 years, approximately 95% of childhood cancer survivors (CCS), will develop significant metabolic disorders, related to the childhood cancer diagnosis and/or its treatment [4]. The most common severe chronic metabolic disorder is cardiovascular disease [5], usually presenting after a varying latency period. Therefore, novel biomarkers are required to identify metabolic abnormalities in the early stages of the disorders.

Irisin, a hormone-like myokine-adipokine, secreted mainly from skeletal muscle during exercise and fasting, as well as from the adipose tissue [6,7], plays a crucial role in the regulation of energy homeostasis. It is considered one of the modulators of adipocyte metabolism, as it induces the browning process of white adipose tissue, and increases thermogenesis. Since its description, irisin has been associated with several metabolic diseases, i.e., type 2 diabetes mellitus, obesity, cardiovascular disease, polycystic ovary syndrome and metabolic dysfunction associated steatotic liver disease. Furthermore, irisin has been related to neurological and metabolic bone diseases [8,9].

Irisin may also be involved in cancer proliferation and migration of cancer cells via different signaling pathways and molecular processes [10]. Inhibitory effects of irisin in the proliferation, migration, and invasiveness of cancer cells have been noted in many studies. A preclinical study of the effect of doxorubicin in mice, showed that irisin was affected by doxorubicin and might have played a role in doxorubicin cardiotoxicity [11]. In another study, the FNDC5/irisin pathway has been demonstrated to enhance cardiac function and aerobic fitness in mice with radiation-induced heart disease [12].

Many studies have also attempted to determine the role of irisin in cancer patients, suffering many types of malignancies, i.e., breast, lung, gastrointestinal, reproductive tract, and bone cancers, as well as its potential role in cancer therapy. The results of these studies are conflicting, as irisin concentrations were increased or decreased in patients with the same or different type of malignancies compared with controls [13,14,15,16,17,18,19,20,21,22]. Similarly, most of the studies have found increased concentrations of irisin in cancer tissues [15,23,24,25], while few studies have demonstrated an opposing trend [16,24,26]. It is not clear, whether the altered expression of irisin, observed in tumor tissue is the cause of tumorigenesis, or a compensatory mechanism to combat tumorigenesis [19], implying that more in vivo studies are required to render irisin as a marker for cancer diagnosis, prognosis or treatment.

Only a few studies have examined the role of irisin in cancer survivors post-treatment. A study evaluating clinical and genetic predictors of weight changes in breast cancer survivors, found that serum irisin concentrations did not correlate with BMI at baseline. On the other hand, rs726344, a FNDC5 *SNP* (single-nucleotide polymorphism), known for altering insulin sensitivity, was significantly associated with weight change at 18 months of breast cancer diagnosis in univariate, but not multivariate, analysis [27]. Additionally, irisin concentrations have been studied in relation to movement behaviors and bone turnover markers in young CCS; however, no correlation with either bone turnover markers, or physical activity variables was found [28]. Interestingly, to the best of our knowledge, there are no studies of the association of circulating irisin to the metabolic and reproductive profile of CCS, while, to date, there is only one study of the relation of irisin to bone health in CCS. Therefore, the aim of this study was to examine irisin concentrations in children and adolescents, CCS (vs. controls) and their association with metabolic, bone and hormonal parameters.

## 2. Materials and Methods

### 2.1. Study Design—Setting

This was a pilot, case–control study, conducted at the Center for Adolescent Medicine and UNESCO Chair in Adolescent Health Care, in collaboration with the Pediatric Hematology Oncology Unit of the Fist Department of Pediatrics, Medical School, National and Kapodistrian University of Athens, at the tertiary Aghia Sophia Children’s Hospital, the largest pediatric hospital in Greece. The study was conducted from July 2017 to February 2022, in agreement with the Helsinki Declaration for human studies and was approved by the Ethics Committee of the Aghia Sophia Children’s Hospital (22129/29-09-17). Eligible participants were assessed on the basis of the inclusion and exclusion criteria and were informed about the procedures and the aim of the study. Written informed parental consent was obtained for all participants.

### 2.2. Study Participants

Study participants included children and adolescents CCS, who were followed up the Pediatric Hematology Oncology Unit of the Fist Department of Pediatrics, as well as gender and age matched controls, who presented for routine annual healthcare visits to the Centre for Adolescent Medicine and UNESCO Chair in Adolescent Health Care of the First Department of Pediatrics.

CCS and controls between 8 and 18 years of age without intellectual disability or psychiatric condition were eligible for inclusion in the study. CCS should be at least 6 months post-treatment for hematologic malignancy or solid tumor, in the absence of another chronic disease, such as intellectual disability, psychiatric conditions and eating disorders, whereas controls should be clinically healthy and under no medication.

### 2.3. Variables—Procedures

All participants underwent a detailed physical, biochemical and hormonal assessment, as well as bioelectrical impedance analysis (BIA).

#### 2.3.1. Anthropometric Data

At enrollment, medical history and anthropometric data were recorded. All body measurements were obtained by the same physician. Body mass index (BMI) along with BMI z-score were calculated, as the ratio of body weight (in Kg) to the square of height (in m^2^). Subjects were classified as having obesity or overweight, according to the International Obesity Task Force (IOTF) criteria [29].

The pubertal developmental stage was determined according to Marshall and Tanner stages. Pubertal development was categorized into two groups based on breast and genital stages (prepubertal, boys with genital stage I and girls with breast stage I; and pubertal, boys with genital stage ≥ II and girls with breast stage ≥ II).

Blood pressure was measured in each participant at the right arm after a 10 min rest in the supine position with an oscillometric device. Blood pressure was measured twice, 5 min apart, and the lowest value of systolic and diastolic blood pressure measurements was recorded. The cuff size was adapted to the length and circumference of each participant’s upper arm and was as large as possible to prevent the elbow skin crease obstructing the stethoscope [30].

#### 2.3.2. Blood Parameters

Blood samples were collected from each participant in the morning at 8:00–9:00 am after an overnight fast, and between the 2nd and 5th day of a spontaneous bleeding episode in the post-menarcheal female participants. Biochemical tests and serum hormones were analyzed immediately, whereas the supernatant serum was kept frozen at −80 °C pending irisin analysis. Serum concentrations of irisin were measured with the use of the Irisin (Human) Elisa Kit with sensitivity of 100 pg/mL. The intra-assay and inter-assay precision ranged between 4 and 6% and 8 and 10%, respectively. The homeostatic model assessment for insulin resistance (HOMA-IR) was used to assess insulin resistance and was calculated according to the formula HOMA-IR = fasting glucose (in mg/dL) × fasting insulin (in μIU/mL)/405. Insulin sensitivity, was estimated by the quantitative insulin sensitivity check index (QUICKI) using the formula QUICKI = 1/[log(fasting insulin in μIU/mL) + log (fasting glucose in mg/dL)] [31].

#### 2.3.3. Body Composition Analysis

The body composition (fat mass, free fat mass, adipose tissue, total body water, extracellular body water, intracellular body water, IMAT, body density, bone) of each participant was determined using the BIA method with the use of a multi-frequency bioelectrical impedance technology device developed by BioTekna (BIA-ACC), Marcon, Venice, Italy. [32].

#### 2.3.4. Statistical Analysis

This was a pilot study [33]. A significance level of *p* < 0.05 was set for all analyses. IBM SPSS Statistics, version 29 (IBM Corp. Released 2023. IBM SPSS Statistics for Windows, Version 29.0.2.0 Armonk, NY, USA: IBM Corp) was employed for statistical analysis. Descriptive statistics (means, standard deviations, absolute and relevant frequencies) were calculated for all study variables. The normality of continuous variables was assessed using the Kolmogorov–Smirnoff, Shapiro–Wilk test and visual inspection of Q–Q plots. Depending on the normality of the data, Pearson’s or Spearman’s correlation coefficients were used to examine associations between continuous variables. Independent samples Student *t*-test and Mann–Whitney U test were performed to compare group differences. To examine predictors of the outcome variable, multiple linear regression analysis was employed, entering relevant independent variables simultaneously.

## 3. Results

### 3.1. Characteristics of Study Participants

A total of 59 children and adolescents aged 8–18 years (mean age ± SD 12.84 ± 2.9 years; 27 males and 32 females; 10 prepubertal and 49 pubertal) were included in the study. The group of CCS included 36 participants (mean age ± SD 13.27 ± 2.84 years; 16 males, 20 females; 6 prepubertal and 30 pubertal), and the control group consisted of 23 participants (mean age ± SD 12.16 ± 2.93 years; 11 males, 12 females; 4 prepubertal and 19 pubertal). The groups were gender, age, Tanner stage and BMI matched (*p* > 0.05). Clinical characteristics, biochemical, hormonal and body composition parameters of the study groups are listed in Table 1. There were no differences in body composition and metabolic parameters between the two groups.

In the CCS’s group, 13 participants were diagnosed with acute lymphoblastic leukemia, 9 participants with Hodgkin lymphoma, 5 participants with non-Hodgkin lymphoma, 3 participants with nephroblastoma, 2 participants with brain tumor, 1 participant with acute myelogenous leukemia and other CCS with other types of cancer (sarcoma, nasopharyngeal Ca). The median (IQR) time from last cancer treatment (chemotherapy ± radiotherapy) was 20.50 (25.50) months.

### 3.2. CCS vs. Controls

**Figure 1:**
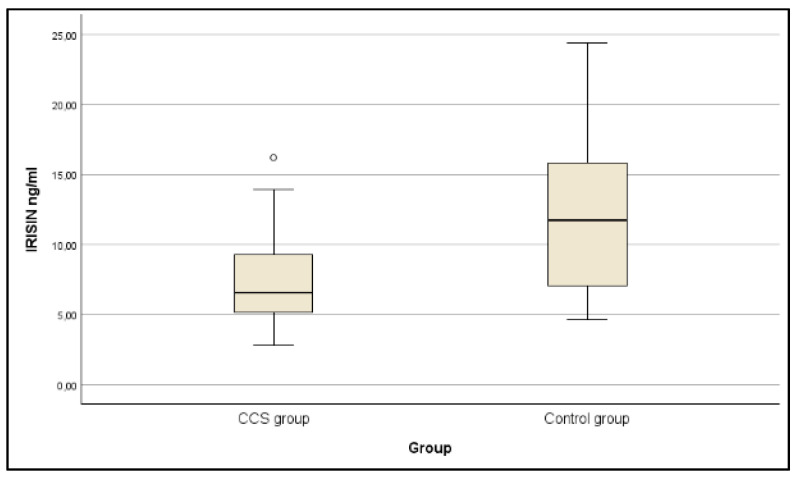
Irisin concentrations in CCS and controls.

Children and adolescents CCS had significantly (*p* < 0.001) lower serum irisin concentrations than controls (Figure 1). They also had significantly lower ALP (*p* = 0.013) and phosphorus (*p* = 0.048) with no differences in other markers of bone metabolism (except for irisin). Platelet count was also lower but without thrombocytopenia (*p* = 0.009).

Albumin and CRP concentrations were higher in the group of CCS vs. controls (*p* = 0.021, *p* = 0.003, respectively). CCS also had higher FSH concentrations (*p* = 0.043), with no statistically significant differences in the concentrations of LH and gonadal steroids between the two groups (Table 1).

### 3.3. Irisin Correlations

In the total study sample (CCS and controls), serum irisin concentrations did not differ significantly when stratified for gender, BMI, WC or Tanner Stage. However, statistically significant negative correlations were found between serum irisin and LH (r_s_ = −0.314, *p* < 0.05), CRP (r_s_ = −0.366, *p* < 0.005), as well as with age (r_s_ = −0.323, *p* < 0.05). Positive correlations were found between serum irisin and ALP (r_s_ = 0.328, *p* < 0.05), as well as with Cl (r_s_ = 0.352, *p* < 0.05) (Table 2).

In the CCS group, positive correlations were found between serum irisin and ApoB, Lpa, as well as with Cl (r_s_ = 0.410, 0.421 and 0.406, respectively, *p* < 0.05) (Table 2).

In the control group, serum irisin concentrations correlated negatively with age (r = −0.476, *p* < 0.05), and positively with PTH (r = 0.542, *p* < 0.005) (Table 2).

No correlations were found between irisin and BIA parameters (Table 2).

### 3.4. Multivariate Analysis

Multivariate linear regression analysis in the total study sample indicated that PTH was the only independent variable affecting serum irisin concentrations (Table 3).

## 4. Discussion

To the best of our knowledge, this is the first study to evaluate irisin concentrations and their relation to body composition, metabolic, bone and reproductive profile, in children and adolescent CCS. We demonstrated significantly lower concentrations of irisin in CCS than matched healthy controls. PTH was the only independent variable that seemed to affect irisin concentrations. Serum irisin concentrations were not associated with the glycemic profile nor with the body composition parameters of the participants. The relation between circulating irisin and components of the metabolic profile of children, adolescents and adults remains unclear. In most studies, irisin is positively associated with indices of adiposity such as BMI, waist circumference, waist-to-hip ratio (WHR) and fat mass, while other studies report a negative correlation or no association at all [7,34,35,36,37,38,39,40,41,42,43,44]. This study did not demonstrate any correlation between irisin concentrations and BMI, WC and body composition parameters (FM, FFM, AT, SM). Although diversity in age and Tanner stage affect BMI and body composition, CCS and controls were matched for those characteristics.

Several studies examining the relation of circulating irisin with the glycemic profile in non-diabetic populations, demonstrated positive associations with serum fasting blood glucose [36,45,46,47,48], insulin [39,47,48,49,50], and insulin resistance [39,47,48,49,51,52]. In contrast, two studies in pediatric populations demonstrated a negative correlation between irisin and glucose [40,53]. In line with the findings of our study, there are several studies reporting no correlation of irisin concentrations with fasting glucose [37,39,44,49,54], insulin or insulin resistance [37,44,53,54].

Regarding diabetic populations, most studies demonstrated lower circulating irisin concentrations in patients with T2DM or prediabetes than controls [45,55,56,57,58], whereas in an Italian study, irisin concentrations were higher in children and adolescents with type 1 diabetes mellitus (T1DM) than controls [34].

Data regarding irisin and lipid metabolism are also inconsistent. Few studies have reported a positive correlation between irisin concentrations and a pathologic lipid profile, such as increase in triglycerides, total cholesterol and low-density lipoprotein cholesterol [39,48,50,58,59,60]. Irisin has been reported to have both, negative and positive associations with high-density lipoprotein cholesterol (HDL) [39,48,49,61,62,63]. In a Greek study of preadolescents and adolescents born preterm or full-term, serum irisin correlated negatively with their subscapular skinfold and positively with HDL [64]. Nevertheless, in several studies, no association of irisin with lipids has been detected [37,40,44,49,53,65,66,67,68]. We found positive correlations of serum irisin with the markers of cardiovascular risk, ApoB and LpA, only in the CCS group.

In addition to its regulatory role in metabolism, a limited number of studies have suggested irisin as a potential anti-inflammatory agent [69]. C-reactive protein (CRP) is an acute-phase reactant, which increases in response to any inflammatory stimulus and then decreases acutely [70]. Serum concentrations of CRP, and other inflammatory biomarkers are influenced by physical activity via muscle contraction [71], implying a relation to irisin. In this study, irisin exhibited a significant, yet negative correlation to CRP, in accordance with the results of Hou et al., who assessed the relationship between circulating irisin and endothelial function in lean and obese subjects [72]. On the other hand, Buscemi et al. found a significant positive correlation between irisin and high-sensitivity CRP concentrations in the general population [73]; however, in another study conducted in clinically healthy adults, no significant correlation was found. A systematic review and meta-analysis of existent studies found no overall significant correlation between irisin and CRP concentrations, although a significant positive correlation was found for overweight and obese subjects [74].

In our study, serum irisin concentrations correlated negatively with age in the total sample and in the control group but did not differ significantly when the study sample was stratified for gender and Tanner stage. Throughout the current medical literature, the relation between irisin and the hypothalamic-pituitary-gonadal (HPG) axis has been largely investigated with various outcomes. As far as sex is concerned, there are studies showing higher serum concentrations of irisin in females than in males [53], either in minor or adult populations [65], while other studies found no difference between the sexes in adults [75,76]. In puberty, irisin levels increase significantly with the activation of the HPG axis [39,77]. During the menstrual cycle changes in irisin concentrations can be observed with increase in irisin during the luteal phase by approximately 25% compared with the follicular phase [78]. Irisin seems to have dual effects on GnRH and gonadotropins. More specifically, research shows that irisin could promote the expression of FSH and LH [79,80,81] and on the other hand, irisin could compete with GnRH to inhibit the secretion of FSH and LH [79,81,82,83,84]. These effects occur at the same time and interact with each other, and changes in circulating hormonal levels are achieved when one activity is dominant [85]. In accordance with the above, data show a positive relation between irisin and E2 levels [36,81,82], which could be justified by the expression of gonadotropins, or because of the effect of irisin on ovarian aromatase activity [86,87]. As for androgens, results are inconsistent, presenting either a positive [88,89] or a negative [90,91,92] correlation between circulating irisin and testosterone concentrations, or no significant correlation overall [36]. A negative relation of irisin with LH concentrations in total sample, but no correlations with FSH, E2 or androgens, were found in the present study.

Numerous studies have shown significant correlations between irisin and bone health [93,94,95,96,97,98,99,100,101,102]. High serum irisin concentrations seem to be positively correlated with bone mass, bone mineral density and bone turnover in healthy adults [103,104,105]. Low serum irisin concentrations may increase the risk of fracture and lead to a series of bone disorders, such as osteoporosis, rheumatoid arthritis and osteoarthritis. Taking into account that irisin can affect the physiological function of bone tissue cells through multiple signaling pathways, it has been proposed to serve as a potential therapeutic target for bone diseases, and to promote bone fracture healing [106]. A few studies investigated the role of irisin in bone metabolism during childhood and adolescence indicating positive associations of irisin with bone mineral density and bone quality [42,107]. Furthermore, high irisin concentrations seem to correlate with a better glycemic control and bone health in children affected with T1DM [34].

Although, no correlations of irisin concentrations with bone density and bone measured by BIA were found in this study, we demonstrated a positive correlation with ALP in the total sample along with a positive correlation of irisin with PTH in the control group. Noteworthy, PTH seemed to be a strong and independent influence on irisin concentrations in our study. There are studies implying an interplay between PTH and irisin, as both hormones affect bone, muscle, and adipose tissue, apparently in opposite ways [108,109]. Palermo et al. have shown a downregulation of FNDC5 in myotubes under treatment with PTH and a reduction in PTH-r mRNA expression in osteoblasts after recombinant irisin exposure [110]. In the same study, these findings seemed to be confirmed by the significant reduction in irisin concentration in postmenopausal women with primary hyperparathyroidism [110]. Gil-Cosano J et al. investigated the association of movement behaviors with irisin, sclerostin, and bone turnover markers in young pediatric cancer survivors, demonstrating no significant correlation between these hormones and bone turnover markers [28]. However, they demonstrated that reducing sedentary time and increasing physical activity may favor bone formation over resorption in young pediatric cancer survivors via higher levels of bone formation markers (such as ALP) and a positive relation of physical activity with PTH concentrations [28].

This study has some limitations. The sample size was relatively small. The CCS group comprised survivors of different types of cancer who had received various therapies, which may have acted as confounders. Furthermore, physical activity of the participants was not assessed. Noteworthy, Albrecht et al. have questioned data obtained with commercial ELISA kits for irisin [111]. Selection of the most appropriate method for irisin quantification, based on its sensitivity, specificity, and practical applicability is crucial. This study, on the other hand has several strengths, as, to our knowledge, it is the first study to determine irisin concentrations in children and adolescent CCS, and their relation to a variety of metabolic, bone and reproductive parameters. Moreover, BIA was used to objectively measure body composition.

## 5. Conclusions

Serum irisin concentrations differed significantly between children and adolescent CCS and controls and were affected by PTH, strengthening the irisin–PTH interplay hypothesis. Future studies are needed to clarify the potential role of irisin as a bone biomarker of CCS in childhood and adolescence. Taking into consideration that this was a pilot study, further investigation with larger sample sizes is also warranted to explore the relation of irisin with the characteristics of survivors of different types of cancer.

## Figures and Tables

**Table 1:** Demographic, anthropometric, body composition, biochemical and hormonal parameters, and irisin concentrations of study groups (CCS vs. controls).

Parameters	CCS Group	Control Group	*p*
Age	13.27 ± 2.84	12.16 ± 2.93	0.153
Ht (cm)	157.54 ± 15.07	152.83 ± 17.35	0.275
Wt (Κg) **^†^**	59.00 (25.00)	54.00 (23.00)	0.155
BMI (Kg/m^2^) **^†^**	22.58 (6.49)	21.64 (7.24)	0.410
WC (cm) **^†^**	75.50 (16.75)	75.00 (18.50)	0.360
HC (cm)	92.75 ± 10.73	87.96 ± 18.65	0.361
Gender (male)	16 (59.3%)	11 (40.7%)	>0.999
Tanner Stage (I, prepubertal)	6 (16.7%)	4 (17.4%)	>0.999
SBP (mmHg)	110.02 ± 12.40	107.57 ± 12.62	0.480
DBP (mmHg)	63.20 ± 9.27	62.45 ± 9.69	0.776
Pulses (bpm)	78.81 ± 14.37	78.00 ± 13.48	0.840
FFM%	76.74 ± 11.17	76.86 ± 10.51	0.974
FM%	23.26 ± 11.17	23.80 ± 10.44	0.873
SM% FFM	35.54 ± 5.54	35.93 ± 5.27	0.818
AT% BW	29.10 ± 14.00	29.76 ± 13.21	0.878
TBW% BW	52.49 ± 8.26	53.27 ± 8.33	0.761
ECW% TBW	44.97 ± 4.31	44.80 ± 4.52	0.899
ICW% TBW	55.03 ± 4.31	54.53 ± 4.66	0.718
FFM (kg)	44.31 ± 10.24	41.00 ± 10.69	0.317
FM (kg)	15.82 ± 12.13	15.25 ± 12.76	0.882
SM (kg)	16.13 ± 5.77	14.96 ± 5.86	0.528
AT (kg)	19.81 ± 15.20	19.09 ± 16.04	0.881
IMAT (kg)	1.27 ± 0.62	1.29 ± 0.62	0.920
Body Density (kg)	1.04 ± 0.02	1.04 ± 0.02	0.630
Bone (kg)	3.37 ± 0.94	3.20 ± 0.88	0.548
Chol (mg/dL)	167.38 ± 29.22	154.09 ± 30.44	0.113
TG (mg/dL) **^†^**	72.50 (47.00)	58.50 (43.00)	0.089
HDL (mg/dL) **^†^**	57.00 (20.50)	49.00 (15.00)	0.063
LDL (mg/dL)	91.19 ± 23.64	86.65 ± 23.21	0.488
Lp(a) (mg/dL)	6.04 ± 8.00	7.40 ± 6.88	0.286
UA (mg/dL)	4.65 ± 1.15	4.36 ± 0.94	0.329
Apo-A1 (mg/dL)	147.87 ± 27.15	137.05 ± 20.62	0.122
Apo-B (mg/dL)	83.53 ± 18.42	77.86 ± 17.71	0.270
Glu (mg/dL)	83.41 ± 7.54	84.36 ± 6.37	0.627
HbA1c (%)	5.10 ± 0.31	5.13 ± 0.15	0.641
Ins (μUI/mL) **^†^**	11.20 (8.16)	8.55 (6.77)	0.206
HOMA-IR **^†^**	1.78 (1.63)	1.72 (1.37)	0.228
QUICKI	0.34 ± 0.03	0.35 ± 0.03	0.470
Ca (mg/dL)	9.73 ± 0.31	9.68 ± 0.23	0.485
P (mg/dL)	4.31 ± 0.71	4.66 ± 0.47	**0.048**
Mg (mg/dL)	2.08 ± 0.17	2.03 ± 0.27	0.592
ALP (U/L) **^†^**	208.00 (125.00)	293.50 (298.00)	**0.013**
25-OH-D (ng/mL)	24.16 ± 8.37	23.75 ± 6.02	0.844
PTH (pg/mL) **^†^**	34.80 (20.60)	29.90 (13.60)	0.124
Hgb (g/dL)	13.62 ±1.05	13.21 ± 0.76	0.124
Hct (%)	40.26 ± 2.58	39.70 ± 2.51	0.429
PLT (×10^9^/L)	231.89 ± 61.42	276.95 ± 59.56	**0.009**
U (mg/dL)	28.12 ± 9.53	27.6 ± 4.95	0.791
Cr (mg/dL)	0.57 (0.21)	0.59 (0.20)	0.724
AST (U/L) **^†^**	21.00 (9.00)	21.00 (9.00)	0.686
ALT (U/L) **^†^**	16.00 (7.00)	14.00 (9.20)	0.840
γ-GT (U/L) **^†^**	11.00 (5.00)	11.00 (5.00)	0.544
Protein Total (g/dL)	7.20 ± 0.42	7.07 ± 0.51	0.370
Alb (g/dL) **^†^**	4.80 (0.40)	4.55 (0.38)	**0.021**
CK (U/L) **^†^**	99.00 (51.00)	104.00 (64.00)	0.535
LDH (U/L) **^†^**	225.50(65.00)	203.00 (91.00)	0.901
Na (nmol/L)	139.79 ± 1.92	140.79 ± 1.40	0.052
K (nmol/L)	4.34 ± 0.24	4.32 ± 0.25	0.773
Cl (nmol/L)	100.16 ± 2.15	101.4 ± 3.97	0.298
CRP (mg/L)	2.19 ± 3.42	1.40 ± 1.93	**0.003**
T3 (ng/mL)	1.25 ± 0.23	1.34 ± 0.32	0.210
TSH (μUI/mL) **^†^**	2.26 (1.69)	2.07 (1.40)	>0.999
FT4 (pmol/L) **^†^**	14.20 (2.30)	14.20 (3.20)	0.551
FSH (mU/mL) **^†^**	4.55 (4.39)	2.93 (2.88)	**0.043**
LH (mU/mL) **^†^**	3.48 (6.21)	1.80 (3.66)	0.094
E2 (mUI/mL) **^†^**	23.30 (57.00)	23.25 (13.50)	0.443
PRL (pg/mL) **^†^**	8.50 (3.71)	7.82 (3.89)	0.986
SHBG (nmol/L) **^†^**	42.50 (62.70)	39.45 (41.20)	0.613
TESTO (ng/mL) **^†^**	0.24 (2.39)	0.33 (0.89)	0.935
DHEAS (mg/dL) **^†^**	112.00 (121.10)	109.00 (181.90)	0.904
Δ4-Andro (ng/mL) **^†^**	1.00 (1.09)	0.54 (0.90)	0.069
17-OH-PRG(ng/mL) **^†^**	1.06 (1.12)	1.09 (0.75)	0.418
F (μg/dL)	10.20 ± 4.74	12.33 ± 4.06	0.096
IGF-1 (ng/mL)	215.01 ±100.16	206.03 ± 62.93	0.730
Irisin (ng/mL) **^†^**	6.54 (4.12)	11.70 (8.75)	**0.001**

Abbreviations: Ht: Height, Wt: weight, BMI: Body Mass Index, WC: waist circumference, HC: hips circumference, SBP: systolic blood pressure, DBP: diastolic blood pressure, Hgb: hemoglobin, Hct: hematocrit, PLT: platelets, Glu: glucose, U: urea, Cr: creatinine, Alb: albumin AST: aspartate aminotransferase, ALT: alanine transaminase, γ-GT: gamma-glutamyl transferase, ALP: alkaline phosphatase, P: phosphorus, Chol: cholesterol, TG: triglycerides, HDL: high-density lipoprotein, LDL: low-density lipoprotein, UA: Uric Acid, CK: creatine kinase, LDH: lactate dehydrogenase, K: potassium, Na: sodium, Cl: chloride, Mg: magnesium, Ca: calcium, Apo-A1: apolipoprotein A1, Apo-B: apolipoprotein B, Lp(a): lipoprotein a, CRP: C-reactive protein, HbA1c: hemoglobulin A1c, Ins: insulin, HOMA-IR: homeostatic model assessment-insulin resistance, QUICKI: Quantitative Insulin Sensitivity Check Index, T3: Triiodothyronine, TSH: Thyroid-Stimulating Hormone, FT4: free thyroxine, FSH: follicle-stimulating hormone, LH: luteinizing hormone, E2: estradiol, PRL: prolactin, SHBG: sex hormone binding globulin, TESTO: testosterone, DHEA-S: dehydroepiandrosterone sulfate, Δ4-andro: androstenedione, 17-OH-PRG:17-hydroxyprogesterone, F: cortisol, IGF-1: insuline-like growth factor 1, PTH: parathyroid hormone, 25-OH-D: 25-hydroxyvitamin D, FFM: free fat mass, FM: fat mass, SM: skeletal mass, AT: adipose tissue, TBW: total body water, ECW: extracellular water, ICW: intracellular water, IMAT: intramuscular AT. Values are referred to mean, standard deviations (SD) or ^†^ median and interquartile range (IQR). *p*-values were computed after conducting *t*-test or ^†^ Mann–Whitney U test.

**Table 2:** Correlation analysis between serum irisin concentrations and other study parameters.

Parameters	Total sample	CCS Group	Control Group
Age	−0.323 *^,†^	−0.075 **^†^**	−0.476 *
Somatometric			
Ht (cm)	−0.148 **^†^**	0.037 **^†^**	−0.085
Wt (kg)	−0.107 **^†^**	0.080 **^†^**	−0.085
BMI	−0.067 **^†^**	−0.010 **^†^**	−0.012
WC (cm)	0.056 **^†^**	0.206 **^†^**	0.004
HC (cm)	−0.147 **^†^**	0.155 **^†^**	−0.191
SBP	−0.053 **^†^**	0.119 **^†^**	−0.086
DBP	0.066 **^†^**	0.064 **^†^**	0.253
Pulse	0.120 **^†^**	0.090 **^†^**	0.223
BIA			
FFM%	−0.033 **^†^**	−0.069 **^†^**	0.079
FM%	0.032 **^†^**	0.069 **^†^**	−0.159
SM% FFM	−0.092 **^†^**	0.005 **^†^**	−0.338
AT% BW	0.026 **^†^**	0.066 **^†^**	−0.193
TBW% BW	−0.038 **^†^**	−0.077 **^†^**	0.105
ECW% TBW	0.071 **^†^**	−0.001 **^†^**	0.285
ICW% TBW	−0.072 **^†^**	0.001 **^†^**	−0.128
FFM (kg)	−0.071 **^†^**	0.181 **^†^**	−0.273
FM (kg)	0.016 **^†^**	0.123 **^†^**	−0.143
SM (kg)	−0.050 **^†^**	0.150 **^†^**	−0.270
AT (kg)	0.017 **^†^**	0.117 **^†^**	−0.143
IMAT (kg)	0.086 **^†^**	0.133 **^†^**	−0.140
Body Density (kg)	−0.077 **^†^**	−0.018 **^†^**	0.023
Bone (kg)	−0.050 **^†^**	0.147 **^†^**	−0.289
Lipid profile			
Chol (mg/dL)	0.045 **^†^**	0.296 **^†^**	−0.120
TG (mg/dL)	−0.033 **^†^**	0.163 **^†^**	−0.103
HDL (mg/dL)	−0.035 **^†^**	0.107 **^†^**	−0.028
LDL (mg/dL)	0.045 **^†^**	0.306 **^†^**	−0.181
Lp(a) (mg/dL)	0.228 **^†^**	0.421 *^,†^	0.115
UA (mg/dL)	−0.071 **^†^**	0.180 **^†^**	−0.173
Apo-A1 (mg/dL)	0.139 **^†^**	0.258 **^†^**	0.159
Apo-B (mg/dL)	0.111 **^†^**	0.410 *^,†^	−0.115
Glycemic profile			
Glu (mg/dL)	0.186 **^†^**	0.096 **^†^**	0.269
HbA1c (%)	−0.036 **^†^**	0.089 **^†^**	−0.321
Ins (μUI/mL)	−0.109 **^†^**	−0.286 **^†^**	0.270
HOMA-IR	−0.085 **^†^**	−0.222 **^†^**	0.189
QUICKI	0.040 **^†^**		
Hormones			
T3 (ng/mL)	0.104 **^†^**	−0.200 **^†^**	0.337
TSH (μUI/mL)	−0.198 **^†^**	−0.248 **^†^**	−0.119
FT4 (pmol/L)	0.108 **^†^**	0.073 **^†^**	0.385
FSH (mU/mL)	−0.207 **^†^**	−0.041 **^†^**	−0.201
LH (mU/mL)	−0.314 *^,†^	−0.177 **^†^**	−0.300
E2 (mUI/mL)	0.029 **^†^**	0.131 **^†^**	0.027
PRL (pg/mL)	−0.038 **^†^**	0.107 **^†^**	−0.168
SHBG (nmol/L)	−0.060 **^†^**	−0.147 **^†^**	0.063
TESTO TOTAL (ng/mL)	−0.242 **^†^**	−0.149 **^†^**	−0.266
DHEAS (mg/dL)	−0.072 **^†^**	0.052 **^†^**	−0.175
Δ4-Andro (ng/mL)	−0.262 **^†^**	−0.034 **^†^**	−0.271
17-OH-(ng/mL)	−0.080 **^†^**	0.100 **^†^**	−0.108
F (μg/dL)	−0.066 **^†^**	−0.143 **^†^**	−0.335
IGF-C (ng/mL)	−0.051 **^†^**	0.009 **^†^**	−0.110
Bone Metabolism			
Ca (mg/dL)	0.154 **^†^**	0.204 **^†^**	0.337
P (mg/dL)	0.172 **^†^**	−0.030 **^†^**	0.297
Mg (mg/dL)	0.096 **^†^**	0.197 **^†^**	−0.116
ALP (U/L)	0.328 *^,†^	0.150 **^†^**	0.242
25-OH-D (ng/mL)	−0.051 **^†^**	0.061 **^†^**	−0.164
PTH (pg/mL)	0.035 **^†^**	−0.198 **^†^**	0.542 *
Others			
Hgb	−0.189 **^†^**	−0.260 **^†^**	0.104
Hct	−0.075 **^†^**	−0.208 **^†^**	0.318
PLT	0.380 **^,†^	0.194 **^†^**	0.404
U (mg/dL)	−0.038 **^†^**	−0.032 **^†^**	−0.150
Cr (mg/dL)	−0.140 **^†^**	−0.011 **^†^**	−0.242
AST (U/L)	0.131 **^†^**	−0.022 **^†^**	0.422
ALT (U/L)	0.012 **^†^**	−0.219 **^†^**	0.403
γ-GT (U/L)	−0.219 **^†^**	−0.210 **^†^**	−0.186
Protein Total (g/dL)	−0.103 **^†^**	−0.163 **^†^**	0.086
Alb (g/dL)	−0.094 **^†^**	0.022 **^†^**	0.201
CK (U/L)	0.176 **^†^**	0.202 **^†^**	0.357
LDH (U/L)	0.186 **^†^**	0.100 **^†^**	0.377
K (nmol/L)	0.153 **^†^**	0.006 **^†^**	0.434
Na (nmol/L)	0.224 **^†^**	0.265 **^†^**	−0.025
Cl (nmol/L)	0.352 *^,**†**^	0.406 *^,†^	−0.100
CRP (mg/L)	−0.366 **^,**†**^	−0.152 **^†^**	−0.327

Values are referred to Pearson or ^†^ Spearman correlation coefficients. *p* ** < 0.01, *p* * < 0.05.

**Table 3:** Multiple linear regression of irisin with age, LH, CRP, ALP, Apo-B, Lp(a), PTH, PLT.

Variable	β	t	*p*-Value
(Constant)	0.499	0.060	0.952
Age	0.023	0.062	0.951
LH (mU/mL)	−0.352	−1.551	0.131
CRP (mg/L)	−0.404	−1.430	0.163
ALP (U/L)	0.000	0.064	0.950
Apo-B (mg/dL)	0.000	−0.005	0.996
Lp(a) (mg/dL)	0.080	1.569	0.127
PTH (pg/mL)	0.110	2.060	**0.048**
PLT	0.024	1.805	0.081

*R*^2^ = 0.407, *R*^2^_adj_ = 0.249.

## Data Availability

Data are available upon request from the corresponding author.

## Abbreviations

17-OH-PRG	17-hydroxyprogesterone
25-OH-D	25-hydroxyvitamin D
Δ4-andro	androstenedione
γ-GT	gamma-glutamyl transferase
Alb	albumin
ALP	alkaline phosphatase
ALT	alanine transaminase
AST	aspartate aminotransferase
AT	adipose tissue
BMI	Body Mass Index
Ca	calcium
CCS	childhood cancer survivors
Chol	cholesterol
CK	creatine kinase
Cl	chloride
Cr	creatinine
CRP	C-reactive protein
DBP	diastolic blood pressure
DHEA-S	dehydroepiandrosterone sulfate
E2	estradiol
ECW	extracellular water
F	cortisol
FFM	free fat mass
FM	fat mass
FSH	follicle-stimulating hormone
FT4	free thyroxine
Glu	glucose
HbA1c	hemoglobulin A1c
HC	hips circumference
Hct	hematocrit
HDL	high-density lipoprotein
Hgb	hemoglobin
HOMA-IR	homeostatic model assessment-insulin resistance
Ht	height
ICW	intracellular water
IGF-1	insuline-like growth factor 1
IMAT	intramuscular AT
Ins	insulin
K	potassium
LDH	lactate dehydrogenase
LDL	low-density lipoprotein
LH	luteinizing hormone
Lp(a)	lipoprotein a
Mg	magnesium
Na	sodium
P	phosphorus
PLT	platelets
PRL	prolactin
PTH	parathyroid hormone
QUICKI	Quantitative Insulin Sensitivity Check Index
SBP	systolic blood pressure
SHBG	sex hormone binding globulin
SM	skeletal mass
T3	triiodothyronine
TBW	total body water
TESTO	testosterone
TG	triglycerides
TSH	thyroid-Stimulating Hormone
U	urea
UA	uric acid
WC	waist circumference
Wt	weight
